# miRNome of Child A hepatocellular carcinoma in Egyptian patients

**DOI:** 10.3389/fonc.2023.1137585

**Published:** 2023-04-24

**Authors:** Hend E. EL-shqnqery, Rania Hassan Mohamed, Omar Samir, Islam Ayoub, Wael M. El-Sayed, Ahmed A. Sayed

**Affiliations:** ^1^ Department of Clinical Pathology, National Liver Institute, Menoufia University, Cairo, Egypt; ^2^ Genomics and Epigenomics Program, Department of Basic Research, Children’s Cancer Hospital Egypt, Cairo, Egypt; ^3^ Department of Biochemistry, Faculty of Science, Ain Shams University, Cairo, Egypt; ^4^ Department of Hepatopancreato Biliary Surgery, National Liver Institute, Menoufia University, Cairo, Egypt; ^5^ Department of Zoology, Faculty of Science, Ain Shams University, Cairo, Egypt

**Keywords:** next generation sequencing, biomarkers, Hsa-miR-214-3p, hsa-miR-4488, hsa-miR-3178, hsa-miR-3182

## Abstract

**Introduction:**

Hepatocellular carcinoma (HCC) has different etiologies that contribute to its heterogeneity. In regards to the number of HCC patients, Egypt ranks third in Africa and fifteenth worldwide. Despite significant advancements in HCC diagnosis and treatment, the precise biology of the tumor is still not fully understood, which has a negative impact on patient outcomes.

**Methods:**

Advances in next-generation sequencing (NGS) have increased our knowledge of the molecular complexity of HCC.

**Results & discussion:**

In this research, 16 HCC and 6 tumor adjacent tissues (control) of Child A Egyptian patients were successfully profiled for the expression profile of miRNAs by NGS. Forty-one differentially expressed miRNAs (DEMs) were found by differential expression analysis, with 31 being upregulated and 10 being downregulated. Kyoto Encyclopedia of Genes and Genomes (KEGG) pathway enrichment analysis was then conducted on these differentially expressed miRNAs revealing that Sensitivity and specificity analysis showed that hsa-miR-4488, hsa-miR-3178, and hsa-miR-3182 were unique miRNAs as they are expressed in HCC tissues only. These miRNAs were all highly involved in AMPK signaling pathways. However, hsa-miR-214-3p was expressed in control tissues about eight times higher than in cancer tissues and was most abundant in “pathways in cancer and PI3K-Akt signaling pathway” KEGG terms. As promising HCC diagnostic markers, we here suggest hsa-miR-4488, hsa-miR-3178, hsa-miR-3182, and hsa-miR-214-3p. We further urge future research to confirm these markers' diagnostic and prognostic potential as well as their roles in the pathophysiology of HCC.

## Introduction

One of the most prevalent gastrointestinal tumors and the second most common cause of cancer-related death worldwide is liver cancer. By 2030, it is estimated that up to 21.6 million new cases of cancer will be diagnosed each year in less developed regions (1). Hepatocellular carcinoma is a global issue, and local epidemiology data revealed regional variations. In Africa and globally, Egypt has the third- and fifteenth-highest populations of HCCs, respectively. In Egypt, HCC is the fourth most frequent cancer (2). Over a decade, the number of HCC patients doubled (3), and consequently, Egyptian health officials view HCC as the most difficult health issue.

Numerous hospital-based studies have revealed that the prevalence of HCC is increasing (3–7). Possible explanations for the rise in HCC incidence are (1) the construction of diagnostic tools and screening programs (8), (2) the higher chance of cirrhotic patients surviving, which raises the risk of HCC, and (3) raising the hepatitis C virus (HCV) incidence and consequences (4). HCV is considered as the primary risk factor for liver cancer in Egypt, including HCC (9).

MicroRNAs (miRNAs), which are small non-coding RNAs, are ubiquitous and frequently bind to target mRNAs’ 3′ untranslated regions (3′UTR). Mature miRNAs are about 22 nucleotides long (10). MiRNAs play a crucial role in tightly controlled processes such as cell division, proliferation, apoptosis, and metabolism (11). Although miRNAs play critical roles in regulating mRNA expression, their precise functions are not all mechanistic details have been revealed yet. It’s interesting to note that 30% of human genes, many of which have associations with malignancies or are located in unstable regions of the genome, are influenced by miRNAs in terms of how they are expressed (12). Human cancer development has been found to be significantly influenced by miRNAs (13–15). The two distinct categories of miRNAs that have been identified are tumor suppressor miRNAs and oncogenic miRNAs (oncomiRs). Tumor suppressor miRNAs reduce oncogene expression in normal cells, whereas oncomiRs promote carcinogenesis by suppressing the expression of tumor suppressors. cancer-related changes were found that, miR-16 and miR-15 are linked to the commonly targeted chromosomal deletion of BCL2, which is responsible for the anti-apoptotic components (16).

Early detection and treatment of HCC can be accomplished by the use of early screening tools, which improve prognosis and overall survival. Unfortunately, the alpha-fetoprotein (AFP), the most used biomarker for HCC, has a limited sensitivity, which hampers the screening procedure (17). Consequently, it is urgently needed to continue looking for novel biomarkers. Recently, NGS technologies have shown to be a potent new force in the arsenal of cancer geneticists, who support the investigation of cancer genomes at higher resolutions (18). The patterns of miRNAs in diverse malignancies and normal tissues vary, and they are crucial in the beginning and progression of cancer (15). In addition, HCC cell lines show dysregulation of the miR-222 (19). Glycine N-methyl transferase is miR-224’s primary target in humans, and it is crucial for the development of HCC tumors (20). In HCC patients, a 20-miRNA signature is associated with survival (21). The aim of the current exploratory pilot study is to profile miRNA for both HCC tissues and tumor adjacent tissues in the Egyptian Child A population (requires surgical intervention) that can pave the way for noninvasive usage of the key identified miRNA for HCC diagnostics and treatment.

## Materials and methods

### Ethical declaration

Each patient gave their understanding and written consent prior to the study’s execution, and the investigation was carried out in accordance with the Good Clinical Practice (GCP) standards of the International Council for Harmonization of Technical Requirements for Pharmaceuticals for Human Use (ICH). The National Liver Institute’s Ethics Committee gave the current study their seal of approval (NLI IRB protocol number 00187\2020).

### Human subjects

A curative liver resection was performed on a total of 21 HCC patients, 15 men and 6 women, with a median age of 58.5 years (range, 28-74 years), 15 tumor specimens and 6 matched tumor adjacent tissues (cirrhotic safety margin area) used as controls were obtained. From March to May 2020, the study’s participants were HCC stage 0 and they had a surgery at the National Liver Institute, Menoufia University followed Barcelona Clinic Liver Cancer, (BCLC) (**Supplementary Figure 1**). All patients had HCV as etiological factor Child A5, A6, according to Child-Pugh classification, MELD score < 9 without preoperative chemo- and radio-therapy. The metastatic patients were out of resection criteria and were excluded from our study. All patients were negative to HBV screening. All cases were histopathologically confirmed.

### Sample collection and RNA extraction

From the patients, tissue samples of the cancerous tissue and adjacent non-cancerous tissues (minimum, 1 cm^3^) were taken. Both malignant and non-cancerous samples received RNAlater immediately and were kept at 80°C until RNA extraction. Total RNA with the small RNA fractions were extracted by using the miRNeasy Mini kit (cat. # 217004) to extract 0.5 g of tissue using TRIzol, as directed by the manufacturer (Qiagen, Germany). The Qubit RNA HS Assay Kit (Invitrogen, USA; Cat. # Q32852) was used to determine the concentrations of the RNA samples.

### miRNA library preparation and sequencing

NEXTFLEX^®^ Small RNA-Seq Kit (PerkinElmer, USA; Cat. # NOVA-5132-05) was used to extract miRNA profiling. According to the manufacturer’s specifications, 700 ng of pure RNA was utilized for each library preparation input. Ligated libraries were reverse-transcribed, amplified, and given their own special barcode primer. Utilizing a 6% TBE-PAGE gel, DNA fragments ~ 150 bp (miRNA sequences plus 3′ and 5′ adaptors) were detected and subsequently processed in a 300 ul elution buffer for purification. Equimolar amounts for each final library were pooled at a final concentration of 4 nM cDNA. The Qubit dsDNA HS Assay (Thermo Fisher Scientific, USA; Cat. # Q33230) was used to measure concentration, and the Bio analyzer DNA assay (Agilent, USA; Cat. No.: 5067-1504) was used to examine the size distribution of the pooled library. At the Genomics and Epigenomics Research Program (GERP) in CCHE 57357, Egypt, a single flow cell of the Illumina MiSeq (Illumina, Inc., USA) was used to sequence the final pooled library for about 2693 miRNAs using 75-bp single-end reads.

### Raw reads processing

**Figure 1:**
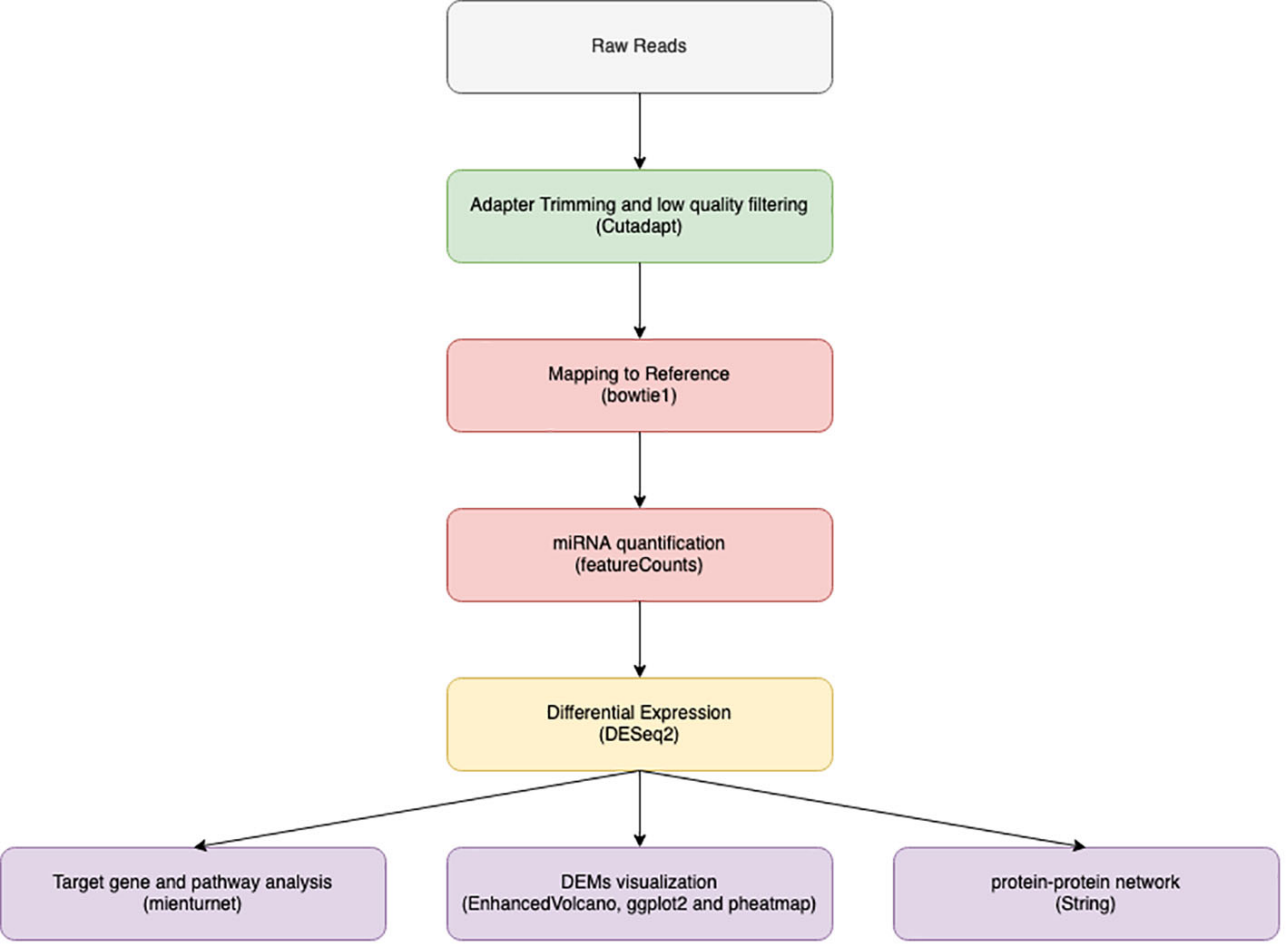
Illustrate data analysis work flow from data filtration through adaptor trimming by cut adapt, alignment using bowtie 1 and finally miRNA quantification using feature count to be used in downstream analysis by DEseq2.

Tools based on Unix were used to analyze the data. as illustrated in **Figure 1**. Raw reads quality inspection was performed using FastQC (22). Trimming the adaptor sequence “TGGAATTCTCGGGTGCCAAGG” with Cutadapt (23) was done after eliminating four bases from both read ends. Only reads between 15 and 28 nucleotides in length were kept out of the processed reads. FastQC was used to evaluate the quality of the filtered reads, and MultiQC was used to compile the results (24).

### Reads mapping and miRNA quantification

The human genome reference GRCh38 (accession number GCA 000001405.29) was used as a reference for bowtie 1 alignment of filtered data. We used the default Bowtie 1 (25) parameters without the mismatch option turned on. MiRNA coordination supplied from the miRBase database (26) was utilized to quantify the mapped reads using feature counts (27).

### Differential expression of HCC miRNA

DESeq2 (28) was used to perform differential expression (DE) analysis on collected counts package in R V4.1.2. DESeq2 was used to compare control and HCC patients after data normalization. DESeq2 includes an internal normalization step for reads per millions. which each miRNA geometric mean (a pseudo-reference sample) was calculated across all samples. Then, for each miRNA in each sample, the miRNA count was divided by the calculated mean. Next, the median (size factor) of these calculations in each sample is calculated. Finally, each raw count is divided by the calculated factor, and the normalization count is generated. This normalization technique considers the differences in sequencing depth.

Selection criteria for differentially expressed miRNAs were > 1.5 log2 fold change and an adjusted *p value (Adj. p)* value < 0.05. All expressed miRNAs values and *adj. p-value* were visualized in volcano plot using Enhanced Volcano (29) and differentially expressed miRNA (DEMs) were illustrated by lollipop using ggplot (30) R packages. Then heat-maps of normalized read count of DEMs were created using pheatmap (31) R package. Finally shared and unique DEMs were illustrated by Venn diagram using Venny (32).

### DEMs target prediction and network inference

Target genes and pathways analysis of DEMs were performed using mienturnet (33) and KEGG (34), the maximum number of interactions and enriched pathways according to miRTarBase (35) were displayed in a bubble plot using ggplot with false discovery rate (FDR) <0.05 and *p value <*0.05. STRING V11 (36) was used to create the protein-protein association network of enriched genes. Finally, a miRNA – target gene network was constructed using miRNet (37) with selecting Liver related data only and Degree filter.

### Statistical analysis

Microsoft Excel 2016 and the social science statistical programme IBM SPSS Statistics for Windows, version 26 (IBM Corp., Armonk, N.Y., USA) were used to analyze the data *P values* < *0.05* were classified as statistically significant for non-normal variables, which were summarized as a median with 25 and 75 percentiles. The Mann-Whitney U test was applied to non-normally distributed variables to compare the median between groups. The receiver operating characteristic (ROC) curve was used to evaluate the study miRNAs’ diagnostic performance. The miRNA selective criteria for ROC curves were based on unique miRNAs either in samples or in control only. As a measure of prognostic test performance accuracy, the area under the curve (AUC) was computed. The greatest combined sensitivity and specificity was used as the cutoff for a group of the study’s diagnosis.

## Result

### Sequencing data and differential expression miRNA analysis

**Figure 2:**
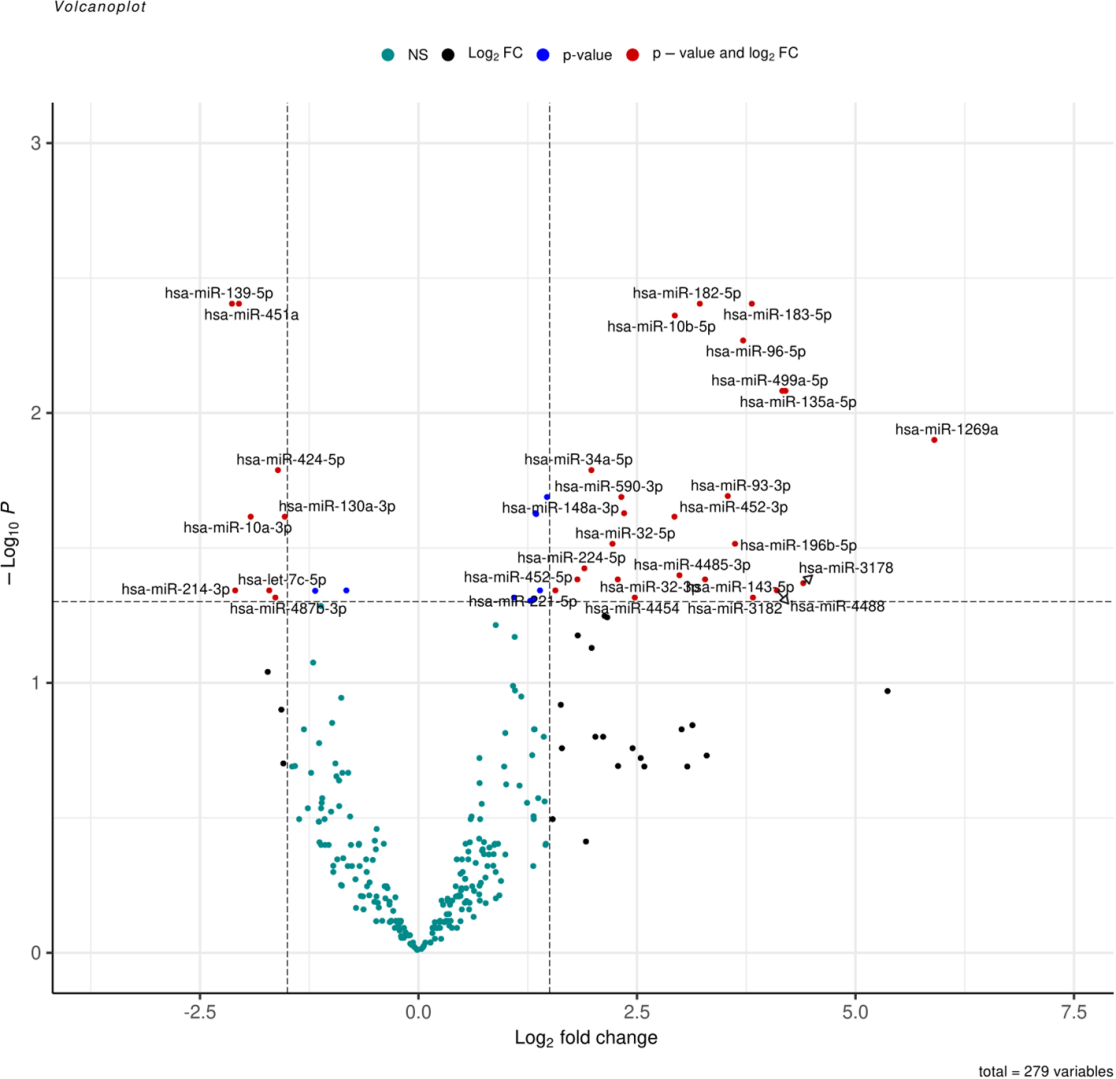
Volcano plot represent all expressed miRNA in samples, log2(fold change) is plotted against-log10(p-value), where p-value is from differential miRNA expression test. The vertical dash line represents fold change of >1.5, while the horizontal dash line represents the p-value <0.05. Red dots denote miRNAs that meet both FC >1.5 and p-value <0.05 criteria, while blue dots meet p-value <0.05 but not FC <1.5, and black dots meet FC > 1.5 but not p-value <0.05 criteria.

**Figure 3:**
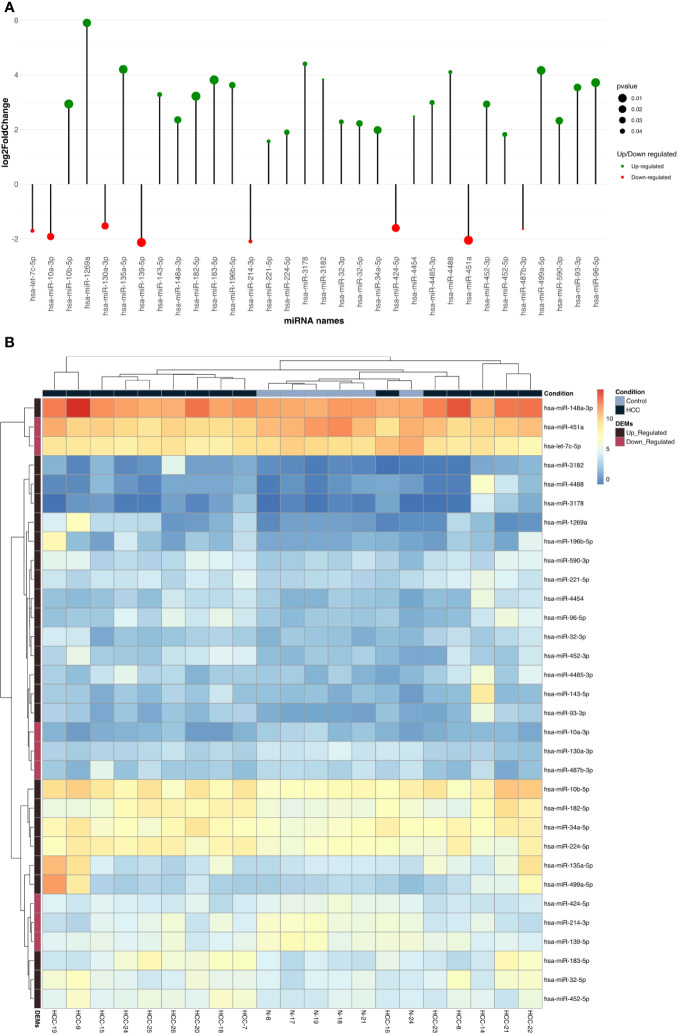
**(A)** Lolliplote represent up and down differentially expressed miRNAs based on log2fold change and *P value*, it showed 23 up regulated with 8 down regulated DEMs. Green circles for up regulated DEMs and Red circles for down regulated DEMs. The circle size reflects *P value*. **(B)** The figure represents heat maps of differentially expressed miRNAs in HCC tissue and tumor adjacent tissue as control (HCC for HCC patient tissues samples and N for tumor adjacent cirrhotic tissue samples) the key representing HCC samples with up or down DEMs. Black square upper the heat maps represent HCC samples and the blue one represent tumor adjacent tissue samples. The black square left the map represent up regulated DEMs and purple one for down regulated DEMs.

**Figure 4:**
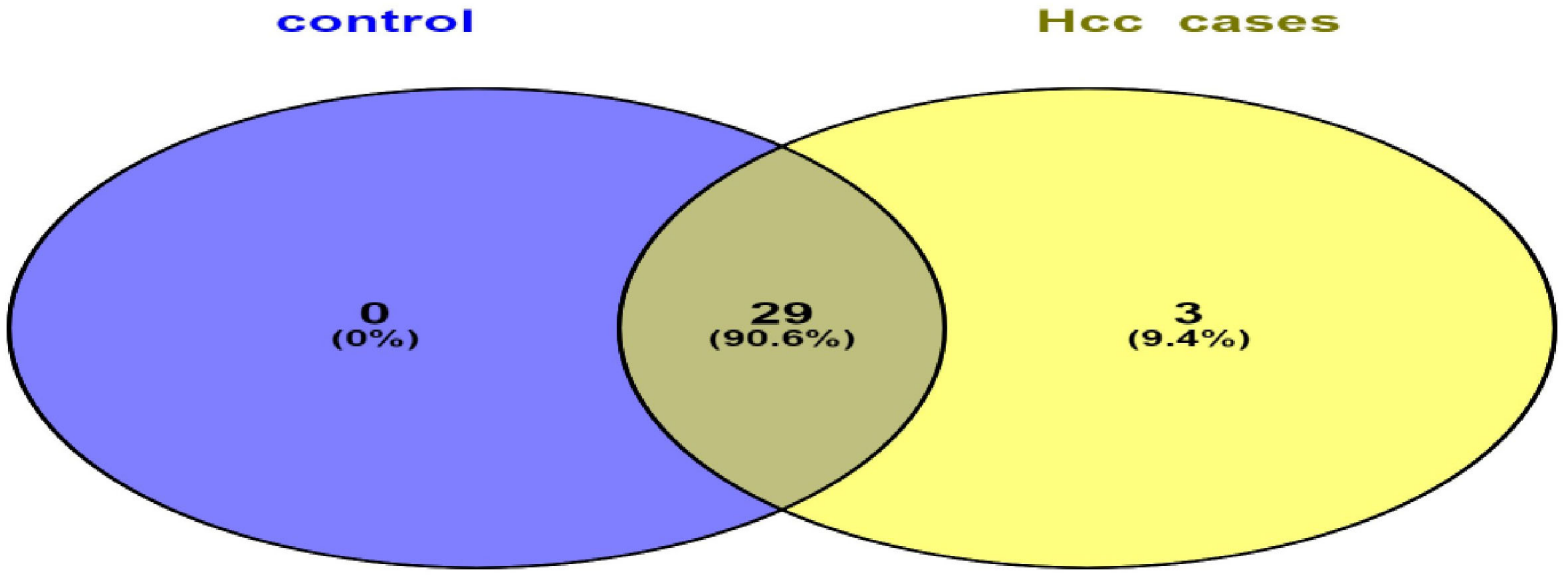
Venn diagram represents shared and unique miRNAs between HCC group and control group. Illustrates that HCC group have unique 3 DEMs and shared 29 DEMs with control group.

Liver tissues from 21 HCC patients were used for library construction yielding, 0.95 million mean reads of samples count (**Supplementary Table 1**). A total of 279 miRNAs were discovered using the predetermined screening conditions, as shown by a volcano plot (**Figure 2**). In order to identify differentially expressed miRNA (DEMs) between tumor and tumor adjacent tissues, all miRNA expression data were imported into the DEseq2 software. Thirty-two DEMs were identified by adjusted P value (*adj. p)* values <0.05 and >1.5 for log2 fold change criteria for up and downregulated miRNAs. Lolliplote clearly shows that 32 miRNAs were retrieved, of which 24 DEMs were upregulated and 8 DEMs were down regulated (**Figure 3A**). The carcinoma samples could be distinguished from the control samples using the heat map clusters of DEMs (**Figure 3B**). Finally, the shared and unique differentially expressed miRNAs between HCC cases and controls were shown using a Venn diagram, to obtain 3 upregulated DEMs; hsa-miR-4488, hsa-miR-3178 and hsa-miR-3182 were represented uniquely in HCC group (**Figure 4**). Our data were compared with 3 different miRNAs datasets. TCGA was based on RNA-Seq technology on American population and sample size was 412 tissue samples. The study was compared HCC samples tissues with normal tissues. GSE 10694 datasets are microarray-based for Chinese population with sample size was 156 containing HCC samples, corresponding noncancerous liver tissue and normal liver tissue. Finally, GSE 6857 dataset also was used as microarray based for American population with 481 samples; HCC tissue and normal tissues. All the mentioned data are merged and clustered with our data and represented by Heatmap (**Supplementary Figure 2**). Moreover, we found that 5 DEMS in our study out of 7 found in OncomiR Cancer database were shared (miR-183, miR-96, miR-10b, miR-224 and miR-424). OncomiR Cancer database study was depending on HCC tissues and normal tissues.

### Prediction of miRNA target genes and enrichment analysis of those genes

**Figure 5:**
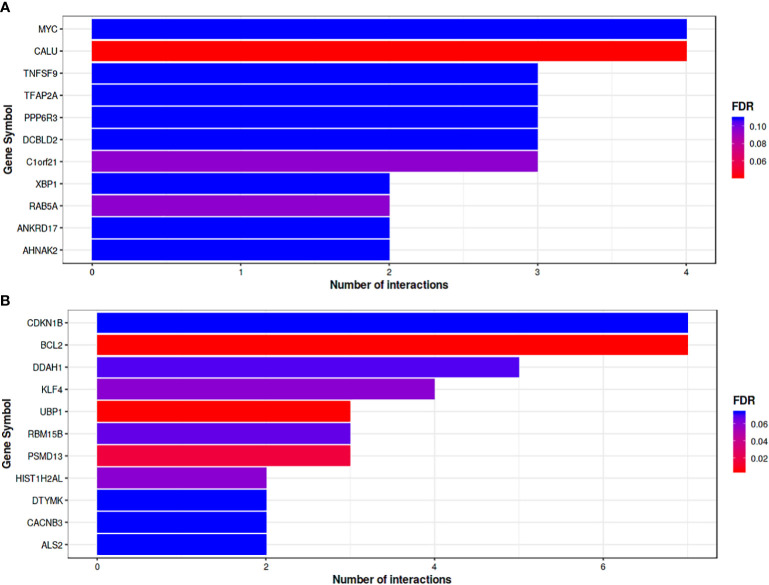
**(A)** Bar plot where the Y-axis refers to the top 11 target genes resulted from the enrichment. Analysis of down-regulated DEMs, while the X-axis represents the number of miRNAs targeting them. The color code reflects the FDR value increasing from red to blue. **(B)** Bar plot Represents top target genes for up-regulated DEMs. Gene symbol was represented by horizontal Axis and vertical Axis was used for number of interactions. Colors reflect FDR value form Red to Blue.

Functional annotation by miRTarBase based only on experimentally validated not predicted targets was used to obtain up and down regulated DEMs, showing that the 8 down regulated DEMs targeting 199 genes with top 11 hub genes illustrated in (**Figure 5A**). Based on the quantity of interactions, *P value <0.05* and FDR, the hub genes were chosen. The two genes that were most prevalent were MYC and CALU. The top 11 hub genes for the 23 up-regulated DEMs that target 866 genes are shown in (**Figure 5B**), which were selected based on the same bases. *CDKN1B* and *BCL2* were the highest down regulated represented genes.

**Figure 6:**
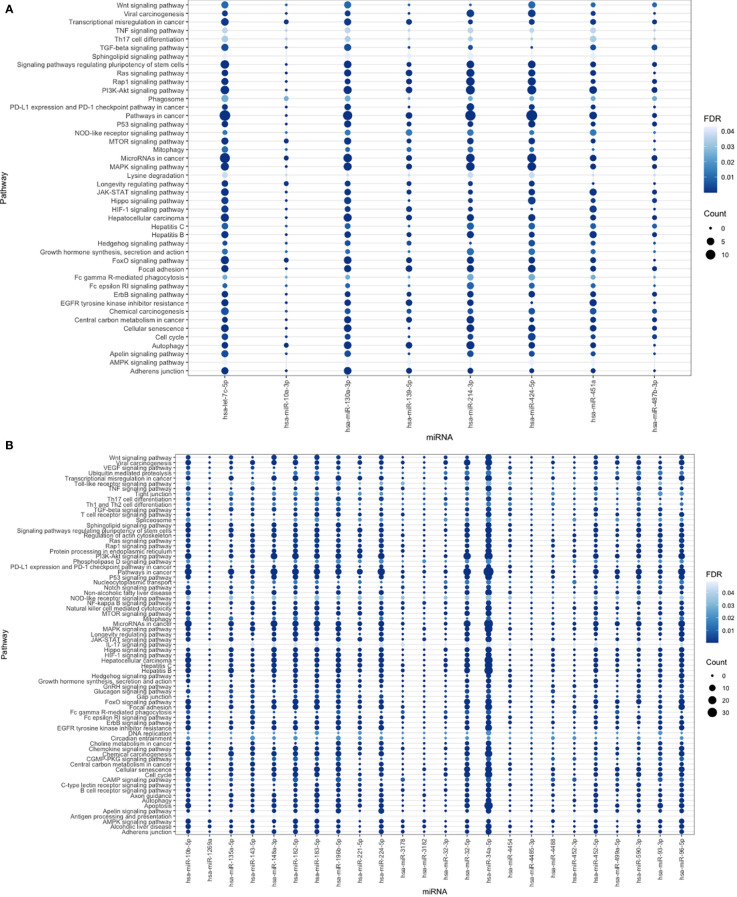
**(A)** The bubble plot represents miRNA-down regulated pathways with false discovery rate (FDR <0.05) indicated by degree of blue color and for number of interacted genes was represented by circle size. **(B)** Bubble plot for miRNA-up regulated pathways with number of interacted genes represented by circle size with FDR <0.05 represented by blue color degree.

The KEGG database was searched using the retrieved DEMs and genes, the enriched pathways filtered out based on *P value < 0.05* and FDR < 0.05 and extensively studied to select the most HCC correlated pathways. The most significantly enriched pathways were 44 pathways (**Figure 6A**). Downregulated DEMs were strongly linked to the FOXO signaling pathway, the cell cycle, and transcriptional dysregulation in cancer, which were found to be crucial processes in the pathogenesis of HCC, according to KEGG analysis. In case of up regulated DEMs, the pathways filtered out based on the same previous criteria to obtain 73 significantly enriched pathways (**Figure 6B**), which illustrates that genes were significantly related to ubiquitin-mediated proteolysis, VEGF signaling pathways, Tight junction, viral carcinogenesis, AMPK signaling pathway among other processes. Moreover, the FOXO signaling pathway, P53 pathway, pathway in cancer, miRNAs in cancer, and hepatocellular carcinoma pathway all had the highest proportion of upregulated and downregulated enriched target genes. Many enriched pathways, which are confirmed to have important roles for HCC development are common for up-and down-regulated miRNA targets, and this supports the accuracy of our analysis.

### Microrna regulatory networks and their target genes

#### miRNA - target genes network

**Figure 7:**
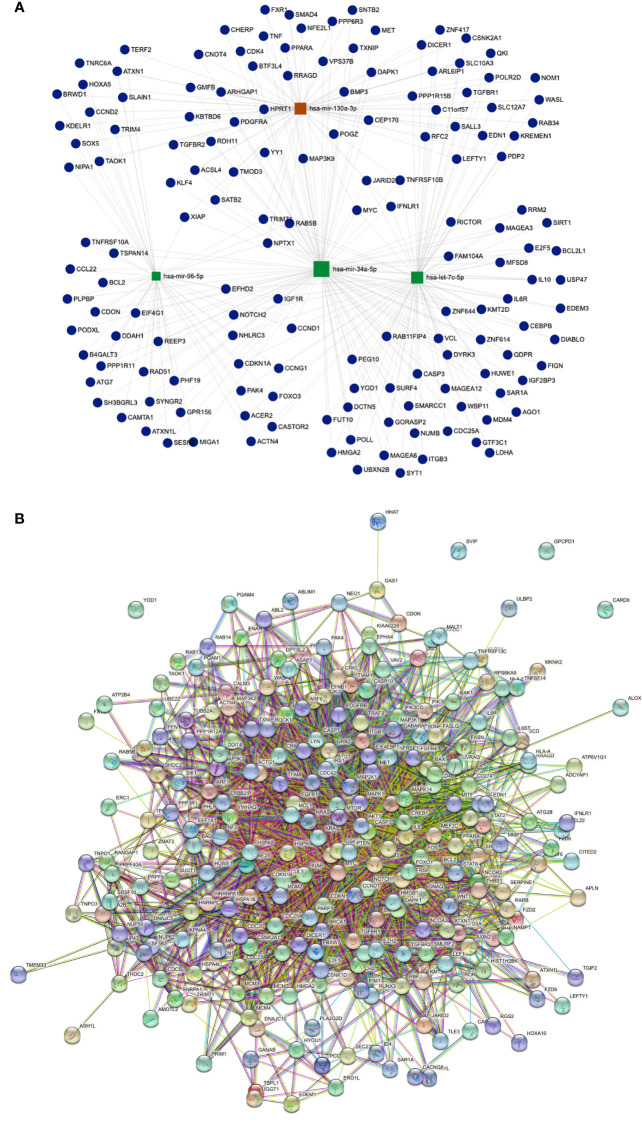
**(A)** Illustrates miRNA-gene interaction network for up and down DEMs, represent 4 hub genes based on the number of interactions. Red color for the highest number of interactions. **(B)** string Protein- Protein interaction network for HCC-related Proteins. Showing high interaction with each other.

A target regulatory network comprising 17 DEMs and their targets implicated in 186 KEGG pathways was created using miRTarBase and KEGG target gene annotation. There are 152 genes and four main miRNAs in the network, depending on the degree of interaction (**Figure 7A**). The top 4 miRNAs were found to target pathways in cancer and PI3K-AKT signaling pathway. Moreover, hsa-miR-130a-3p and hsa-let-7c-5p were enriched in hepatocellular carcinoma, FOXO signaling pathway, and cellular senescence pathways. Likewise, hsa-mir-34a-5p and hsa-mir-96-5p targeted focal adhesion and apoptosis pathways. Interestingly, we revealed that hsa-mir-130a-3p has the most target genes.

#### Protein–protein interaction network

To investigate the relationships and interactions between the HCC-related proteins, the PPI network was built. Out of 1069 proteins, 1061 had strong interactions with one another to form a cluster (**Figure 7B**).

### ROC curve analysis for miRNA in HCC and control tissues

**Figure 8:**
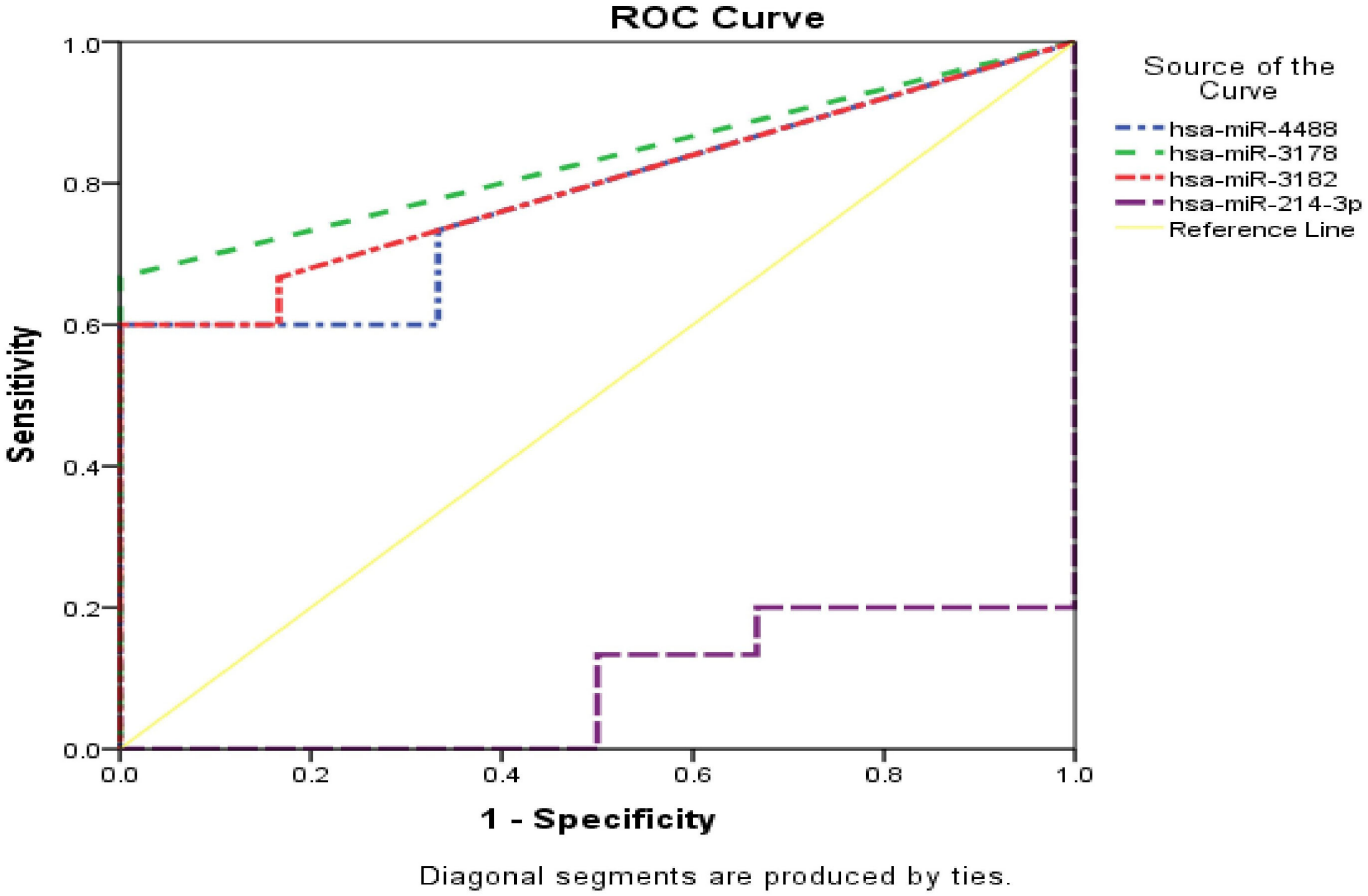
Combined ROC curve for unique miRNA both HCC group and control group. vertical axis represent sensitivity, for specificity horizontal axis was used. Yellow line represent reference, dotted lines with blue, green, red and violet colors represent miR-4488, miR-3178, miR-3182 and miR-214-3p respectively.

Normalized counts of the selected miRNAs according to the selective criteria mentioned previously in the methods section, were used to investigate diagnostic and prognostic potentials for miRNAs by (ROC) curve analysis (**Supplementary Tables 2**, **3**). Hsa-miR-4488, hsa-miR-3178, hsa-miR-3182, and hsa-miR-214-3p all shown strong discriminatory power, hsa-miR-4488 (AUC 0.778, sensitivity (Sn) 73.3%, specificity (Sp) 66.7% with *P* value 0.050 and false positive rate (FPV) 33.3%), hsa-miR-3178 (AUC 0.833, Sn 66.7%, Sp 100% with *P value 0.020* and FPV 0.0%), hsa-miR-3182 (AUC 0.794, Sn 66.7%, Sp 83.3% with *P value 0.039* and FPV 16.7%) and hsa-miR-214-3p (AUC 0.911, Sn 100%, Sp 80% with *P value 0.004* and FPV 20%).All significant miRNAs were combined and represented in (**Figure 8**). However, hsa-miR-1269a was found as insignificant miRNA (AUC 0.728, Sn 53.3%, Sp 100.0% and *P value 0.111*).

## Discussion

The incidence of hepatocellular carcinoma (HCC), the third most prevalent cancer-related cause of death worldwide, is rising (38). Infection with the hepatitis C and B viruses (39), alpha-1-antitrypsin deficiency (40), heavy alcoholism (41) cirrhosis (42), hemochromatosis (43), are risk factors associated with HCC. The prognosis for individuals with HCC remains dismal despite significant advancements in the treatment of the disease, primarily due to the high prevalence of diagnosis at advanced stages (44). Due to their crucial involvement in the genesis and prognosis of cancer, miRNAs have recently gained attention as potential biomarkers (45). In HCC, miRNAs have been studied as biomarkers (46). MiRNA dysregulation in HCC has been documented in earlier research. Furthermore, it’s believed that some miRNAs may be able to manage HCC (47). Our study aims to illustrate the miRNA profile in Egyptian HCC patients based on HCC tissue group and tumor adjacent group as control.

Our data analysis revealed that the levels of miR-182-5p and miR-183-5p had significantly increased in HCC tissues by eight and nine folds, respectively. According to our data, miR-183-5p targets *FOXO1, SMAD4*, and *GSK3B* genes involved in transcriptional misregulation in cancer, *FOXO* signaling pathway and alcoholic liver disease. on the other side, we found that miR-182-5p targets number of cell cycle and HCC development genes as *CDKN1A, BCL2*, and *CDKN1B* in vital pathways including HIF-1 signaling pathway, P53 signaling pathway, cell cycle and JAK-STAT signaling pathway (48). The miR-182-183 miRNA cluster, which includes miR-183-5p and miR-182-5p, is situated at 7q31–34 chromosomal regions. Three miRNAs in this miRNA cluster, miR-96, miR-182, and miR-183-5p, all have remarkably similar 5′-seed sequences (49), depending on the type of cancer, acting as either tumor suppressor genes or oncogenes. Rong Yan et al. (50) showed that miR-183-5p overexpression enhanced liver cancer cells in proliferation and migration. This study found that miR-183-5p, which interacts with the 3’-UTR of insulin receptor substrate-1 (IRS-1), was strongly associated to various clinico-pathological characteristics in HCC (50). Recent research demonstrated that miR-183-5p can increase HCC cell proliferation by suppressing the expression of tumor suppressors (including AKAP12, DYRK2, FOXN3, FOXO1 and LATS2) (51) which is agreement with our results. For those with liver cancer, miR-182-5p overexpression has been shown to have diagnostic and prognostic relevance (52). In concordance with our results, in liver cancer tissues and cell lines, miR-182-5p was recently found to be substantially expressed, according to a study (53). MiR-182-5p stimulated liver cancer cell proliferation by downregulating the regulator of calcineurin 1 (RCAN1). RCAN1 is an endogenous protein that inhibits liver cancer cells’ proliferation, migration, invasion, and cell cycle progression (53).

The miR-10b-5p located on the 2q31.1 chromosome is belonging to the miR-10 family (54). HCC cell lines were shown to express hsa-miR-10b-5p at higher levels than control cell lines, according to previous research. This may be related to the invasion and migration of HCC (54–56). This is consistent with our results because it was strongly expressed in the HCC group and seven times more prevalent. We found that miR-10b-5p regulates KLF4 and AKT1 by targeting a number of pathways including chemical carcinogenesis - receptor activation signaling pathways and signaling pathways regulating pluripotency of stem cells. Additionally, a recent study found that the miR-10b-5p levels in the HCC group increased significantly, with a noteworthy rise in the early stage, comparing the diagnostic accuracy of exo-miR-10b-5p to serum AFP, it may exhibit surprising results (57). In HCC, miR-224 was also discovered to have early diagnostic value (58). By controlling PPP2R1B, a downstream target of miR-224-5p, and CPEB3, the miR-224-5p overexpression promoted HCC cell motility and invasion, according to several studies (58–60). In these studies, suggest that miR-224-5p plays a significant role in the occurrence in HCC samples compared to adjacent tumor tissue samples, which is consistent with our findings that miR-224-5P was four times more abundant in HCC tissues with highly represented BCL2, MTOR, and GSK3B genes. These molecules regulate critical pathways such as the JAK-STAT signaling pathway, the PI3K-Akt signaling pathway, apoptosis, and p53 signaling pathway, which are responsible for angiogenesis, stemness, invasion and metastasis (48). Neoteric study supports that miR-183-5p, miR-182-5p, miR-10b-5p, and miR-224-5p have an important role as diagnostic markers for HCC (61). Furthermore, we recorded miR-34a-5p as a hub miRNA with three-fold higher in liver cancer tissues. miR-34a-5p is targeting large number of genes playing important role in cancer development including MYC, MET, cell-cycle related genes CCND1 (cyclin D1), WNT1, MAP2K1, NANOG, TP53, SRC and AKT1 to target mTOR signaling pathway, Wnt signaling pathway, MAPK signaling pathway, p53 signaling pathway among additional crucial pathways, which play crucial role in HCC tumorigenesis, tumor growth and angiogenesis (62). miR-34a-5p is a member of miR-34a family, which is seen as a hotspot for oncological research, which validated experimentally to target p53 gene (63). miR-34 shown to be associated with hypermethylation and tissue regeneration in the liver (64). Although miR-34a expression may be upregulated in cancers, its function in HCC has not yet been thoroughly established. MiR-34a-5p was reported previously in HCC cells as a response to the oxidative stress in HCC cell lines as it showed relation to intracellular oxidative stress status (63). MiR-34a-5p was also downregulated in HCC cell line when compared with normal human hepatocyte cell line (65), which is in disagreement with our findings as miR -34a-5p was 3 folds higher in HCC tissue samples in comparison to its expression level in the adjacent safety margins and that difference may be as a result of difference in the sample type (66, 67).

Recent research suggests that miR-34a-5p plays a significant role in triggering apoptosis by suppressing SNAI1 in lung cancer cells treated with apigenin (68). Furthermore, according to another study, the expression of miR-34a-5p was increased in people with colorectal cancer and linked with both survival and the expression of the clock gene PER2 (69). Therefore, miR-34a-5p may introduce new hope for HCC patients as a target therapy.

Moreover, we identified miR-3182, miR-3178, and miR-4488 as unique DEMs, they were embodied only in HCC tissues with two-folds higher than control tissue samples but for tumor adjacent group miR-214-3p was reported 8 times higher. In concordance with our results (70), showed that miR-214-3p expression was downregulated in primary HCC samples compared with normal liver tissues, and was decreased in HCC recurrence species compared with non-recurrence controls (P = 0.001). Low miR-214-3p level was associated with poor overall survival (OS) (Log rank P = 0.003) and recurrence-free survival (RFS) (Log rank P = 0.007) (70). All selected miRNAs showed no or low false positive values (0-33.3%), which support the high possibility of their usage as biomarkers for Child A HCC patients. miR-3178 and miR-3182 were reported before but in mismatch with our results; MiR-3178 was reported as downregulated miRNA in malignant Hepatic Sinusoidal Endothelial Cells HSECs against healthy HSECs (71), but miR-3182 was recorded in another study used different sample types as it use HCC patients obey Milan criteria, patients without HCC, chronic liver disease patients with or without HCC, alcoholic liver disease and chronic hepatitis C patients) by microarray assay. So the difference may be a result of sample type, detection method and population differences (67). Additionally, the tumor suppressor miR-214-3p was significantly downregulated (8 times) in our HCC group compared to adjacent control tissues. miR-214-3p targets MAP2K3 and MAPK1 to regulate a number of pathways such as FoxO signaling pathway, MAPK signaling pathway and mTOR signaling pathway which is consistent with Wang et al, (2022) study (72).

Moreover, one of the proposed mechanism of action for miR-214-3p in HCC through the overexpression of Hox transcript antisense intergenic RNA (HOTAIR), which suppresses the expression of adhesion-related integrin which ultimately slows the adherence of HCC cells, and increases metastasis (68–74). The downstream target of HOTAIR, Flotillin 1 (FLOT1), a crucial tumor gene that is connected to the invasion and metastasis of malignancies and can be employed as a stable scaffold when enlisting multi-protein complexes (75), makes the HOTAIR/miR-214-3p/FLOT1 an important axis of how HCC invades and spreads (72).

MiR-139-5p expression level was previously reported to be downregulated in HCC patients, and this was linked to a worse prognosis (71– 73). Our results showed that HCC group was four-folds lower in miR-139-5p expression level, which targets IGF1R, MET, and BCL2 that regulate the MAPK signaling pathway, apoptosis, and mTOR signaling pathway. MiR-139-5p has been identified as a crucial player in the initiation and progression of a variety of cancers, including HCC (76). Our expression profile of miR-139-5p in HCC tissue compared to para-carcinoma tissue, was in concordance with data from the GSE54751 HCC cohort and The Cancer Genome Atlas (76). These findings reveal new targets for the therapy of HCC by regulatory connections between miR-139-5p and transcription factor (ETS1) that regulate aerobic glycolysis, proliferation, and metastasis in HCC cells (77–79). miR-451a is recorded as tumor suppressor in HCC cases in many previous studies (80, 81), because it significantly inhibits tumor progression via YWHAZ gene to control the growth and spread of HCC through many targets (82, 83). This is in concordance with our findings where miR-451a was found to be three-folds lower in HCC group. miR-451a could be a promising target for HCC biotherapy as it targets MYC, MAPK1, and BCL2 genes that is actively involved in the HIF-1 signaling pathway, stem cell pluripotency-regulating signaling pathways, and cellular senescence. MiR-487-3p, which is a member of 14q32-encoded miRNAs (84), was found to be downregulated three-fold in our HCC group than adjacent tumor tissues. It targets MYC and THBS1, which participate in the cell cycle, Wnt signaling route, and p53 signaling pathway, among many other pathways, that matched with Geraldo et al. (85), who identified that the miR-487-3p could act as a tumor suppressor. The mechanism of action for miR-487-3p was defined in few cancer types, but not in HCC. Therefore, further research is necessary to clarify the significance of miR-487-3p in HCC cases since it may represent a new target therapy. Also, our study identified miR-424-5p to be differentially expressed and significantly downregulated (three times) in HCC tissues compared to adjacent tissues and that aligned with earlier findings by Zhao et al. (86). As it targets E2F7, MiR-424-5p controls the cell cycle and suppresses proliferation, playing a noteworthy role in the development of HCC (86). Our miRNA gene network analysis showed the connection of miR-424-5p with CDK6, CCND1, and FGFR1 genes which is represented in the Wnt signaling pathway, cell cycle, and FoxO signaling pathway (48).

miR-130a-3p was detected in our study as hub miRNA with three-folds lower in HCC group than control tissues that targets crucial genes as indicated by gene network responsible for HCC progression and metastasis via a number of pathways including cell cycle, Hippo signaling pathway, and mTOR signaling pathway (62). Aligned with our findings Liu etal. (87) find that miR-130a-3p was significantly decreased in cirrhosis but when miR-130a-3p was injected into the liver effectively decreased liver granulomatous inflammation, decreased expression of the tissue inhibitor of metalloproteinase (TIMP), collagen deposition, and increased the expression of matrix metalloproteinase (MMP) which contributed to the dissolution of collagen (87). Which will lead to decrease the chance for HCC initiation and development.

After comparing our data with different datasets used different populations (TCGA, GSE 6857 and GSE 10694) reported in (88), we found that 10 common miRNAs (miR-214, miR-139, miR-424, miR-130a, miR-148a, miR-34a, miR-221, miR-182, miR- 135a, miR-183 and miR-224) were found in at least 2 of 3 datasets (88). We have found expression differences between the included datasets, as result of differences in detection method used, patient’s criteria and population, which may justify the presence of some discrepancies between this study and our data. Moreover, our data offers miRNAs isoforms, as it based on miRNA Seq but for TCGA, GSE 10694 and GSE 6857 were RNA-Seq and microarray based respectively. different populations were used in each study for TCGA and GSE 6857 were performed on Americans, but for GSE 10694 and our study were Chinese and Egyptians, respectively. Because the input material is frequently enriched for short RNAs, the miRNA-Seq varies from other RNA-Seq types. The miRNA-Seq examines tissue-specific expression patterns, disease associations, and isoforms of miRNAs, and to discover previously uncharacterized miRNAs (89, 90). Morgul et al. (67) reported that the miRNA signatures could differentiate the HCC tumor and cirrhotic liver in their underlying disease like alcoholic liver vs. HCV and therefore they could serve for personalized medicine, which confirm that many factors may affect the expression profile and levels of miRNAs (67). While when comparing our data with OncomiRs database found that 5 DEMS (miR-183, miR -96, miR -10, miR -224, miR -424) in our study out of 7 represented in OncomiRs Cancer were shared. OncomiRs database compared the miRNAs profile in HCC patients tissue and normal liver which was noticed in Pvalues numbers for the shared DEMs. The difference in *p* value maybe as a result of sample nature as our samples were HCC tissue stage 0 patients compared with adjacent non tumor cirrhotic area but in OncomiR Cancer, the HCC tissue compared with normal tissues. Moreover, we could not find samples specifically classified as Child A HCC stage.

To the best of our knowledge, this is the first study using NGS technique to figure out miRNA profile in HCC child A Egyptian patients’ tissues and recommend miR-34a-5p, miR-3178, miR-3182, and miR-4488 as diagnostic markers. Additionally, this research illuminates miR-487b-3p’s possible significance in HCC. Further studies may be needed in larger cohort group to validate the identified miRNAs.

## Conclusion

In this study, miRNA profiling for Egyptian hepatocellular carcinoma patients was used to construct a comprehensive miRNA-target genes network and protein-protein interaction network with the illustration of involved significant pathways. 41 differentially expressed miRNAs were identified. Further specificity and sensitivity tests revealed that four miRNAs; hsa-miR-4488, hsa-miR-3178, hsa-miR-3182, and hsa-miR-214 were significantly associated with diagnosis. Therefore, these differentially expressed miRNAs are expected to be potential biomarkers or therapeutic targets for HCC. Further studies are needed for the validation of the identified miRNAs roles in pathophysiology and diagnosis of HCC on large cohort group including higher sample size and different HCC stages

## Data availability statement

The datasets presented in this study can be found in online repositories. The names of the repository/repositories and accession number(s) can be found below: https://www.ncbi.nlm.nih.gov/, SUB12484712.

## Ethics statement

The National Liver Institute’s Ethics Committee gave the current study their seal of approval (NLI IRB protocol number 00187\2020). The patients/participants provided their written informed consent to participate in this study.

## Author contributions

AS and HE-S contributed to the research concept and design. HE-S and RM wrote the manuscript. IA and HE-S provide the samples. OS and RM manage the bioinformatics analysis. HE-S made the sequencing process and wrote the first draft of the manuscript. AS. and WE-S were in responsible of managing and supervising. All authors contributed to the article and approved the submitted version.

## Supplementary material

Supplementary Figure 1 BCLC Barcelona Clinic Liver Cancer, PS performance status, N node classification, M metastasis classification, RFA radiofrequency ablation, TACE transcatheter arterial chemoembolization. https://www.researchgate.net/publication/284798569_Clinical_guideline_SEOM_hepatocellular_carcinoma.

Supplementary Figure 2 Comparison between our data and different data set merged and clustered in heatmap.
