# Supplementary figures and images for: miRNome of Child A hepatocellular carcinoma in Egyptian patients
Source: Front Oncol. 2023 Apr 24;13:1137585. doi: 10.3389/fonc.2023.1137585 (PMC10164962; doi:10.3389/fonc.2023.1137585)

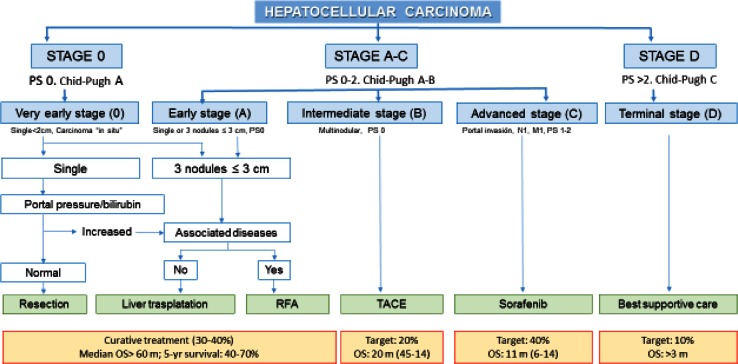

Supplement: Supplementary Figure 1 — BCLC Barcelona Clinic Liver Cancer, PS performance status, N node classification, M metastasis classification, RFA radiofrequency ablation, TACE transcatheter arterial chemoembolization. https://www.researchgate.net/publication/284798569_Clinical_guideline_SEOM_hepatocellular_carcinoma. [file Image_1.jpeg]

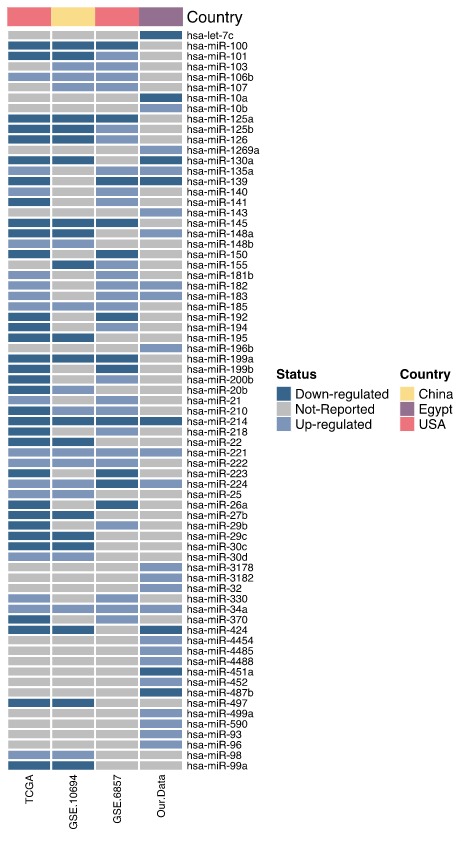

Supplement: Supplementary Figure 2 — Comparison between our data and different data set merged and clustered in heatmap. [file Image_2.jpeg]
